# The pan-genome of *Mycobacterium avium* subsp. *paratuberculosis* (Map) confirms ancestral lineage and reveals gene rearrangements within Map Type S

**DOI:** 10.1186/s12864-023-09752-0

**Published:** 2023-10-31

**Authors:** Rachel Hodgeman, Rachel Mann, Noel Djitro, Keith Savin, Simone Rochfort, Brendan Rodoni

**Affiliations:** 1 Agriculture Victoria, AgriBio, La Trobe University, Bundoora, VIC Australia; 2 School of Applied Systems Biology, AgriBio, La Trobe University, Bundoora, VIC Australia

**Keywords:** *Mycobacterium avium* subsp*. paratuberculosis*, Pan-genome, CAZymes, Prophages, Secondary metabolites, PpanGGolin, IS elements

## Abstract

**Background:**

To date genomic studies on Map have concentrated on Type C strains with only a few Type S strains included for comparison. In this study the entire pan-genome of 261 Map genomes (205 Type C, 52 Type S and 4 Type B) and 7 *Mycobacterium avium* complex (Mac) genomes were analysed to identify genomic similarities and differences between the strains and provide more insight into the evolutionary relationship within this Mycobacterial species.

**Results:**

Our analysis of the core genome of all the Map isolates identified two distinct lineages, Type S and Type C Map that is consistent with previous phylogenetic studies of Map. Pan-genome analysis revealed that Map has a larger accessory genome than *Mycobacterium avium* subsp. *avium* (Maa) and Type C Map has a larger accessory genome than Type S Map. In addition, we found large rearrangements within Type S strains of Map and little to none in Type C and Type B strains. There were 50 core genes identified that were unique to Type S Map and there were no unique core genes identified between Type B and Type C Map strains. In Type C Map we identified an additional CE10 CAZyme class which was identified as an alpha/beta hydrolase and an additional polyketide and non-ribosomal peptide synthetase cluster. Consistent with previous analysis no plasmids and only incomplete prophages were identified in the genomes of Map. There were 45 hypothetical CRISPR elements identified with no associated cas genes.

**Conclusion:**

This is the most comprehensive comparison of the genomic content of Map isolates to date and included the closing of eight Map genomes. The analysis revealed that there is greater variation in gene synteny within Type S strains when compared to Type C indicating that the Type C Map strain emerged after Type S. Further analysis of Type C and Type B genomes revealed that they are structurally similar with little to no genetic variation and that Type B Map may be a distinct clade within Type C Map and not a different strain type of Map. The evolutionary lineage of Maa and Map was confirmed as emerging after M. *hominissuis*.

**Supplementary Information:**

The online version contains supplementary material available at 10.1186/s12864-023-09752-0.

## Background

*Mycobacterium avium subsp. paratuberculosis* (Map)*,* the causal agent of Johne’s disease in ruminants includes three strain variants: Type S (also called sheep strain or Type I/III), Type C (also called cattle strain or Type II) and Type B (also called bison strain) [1–3]. Traditionally these strain types have been differentiated by the presence or absence of a polymorphism in the IS1311 insertion sequence [4] and identified using polymerase chain reaction (PCR) and restriction enzyme analysis (REA) [5]. In more recent times with advances in molecular techniques, whole genome sequencing has allowed for the provision of more in-depth analysis of the genomic relationship between and within species which has resulted in improved diagnostic tests [6].

Type S strains have predominately been isolated from sheep and goats [7], indicating that they have a host preference whereas Type C strains have been shown to have no host preference and have been isolated from a range of domesticated and non-domesticated animals [8]. There is also evidence that there are differences in the Map strain types in their ability to cause disease especially their virulence in different host species. Experimental studies have shown that deer infected with Type C strain established infection in 100% of the animals, while only 69% of deer established infection when inoculated with the Type S strains [9].

The Map-K10 (Type C strain) genome has been completely sequenced and annotated [10] and found to have some homology to *Mycobacterium tuberculosis*, with 75% of the Map genes having counterparts in *M. tuberculosis* [11]. Map is part of the *Mycobacterium avium* complex (Mac) of which there are four subspecies: *M.avium* subsp. *avium* (Maa), *M. avium* subsp. s*ilvaticum* (Mas), *M. avium* subspecies *paratuberculosis* (Map) and *M. avium* subsp. *hominissuis* (Mah) [12]. Through genomic comparisons of the Mac subspecies it is believed that the ancestor of Map is *M. avium* subsp. *hominissuis*, from which insertions, deletions and rearrangements occurred and resulted in the emergence of the pathogen Map [13] and Type S strains are an intermediary between Mah and Type C strains of Map [14].

Genomic studies of Map to date have mainly focused on the expression of genes under different conditions [15, 16] and those genes associated with cell surface proteins and invasion of macrophages [17, 18]. A small number of studies on a limited number of Map isolates have looked at virulence genes [14, 19], but there has been little work focusing on strain specific genes in Map [20].

This study is the first comprehensive analysis of the pan-genome of Map. In this study the genomes of 268 isolates from diverse geographical regions were compared to gain insight into the differences and similarities between Mac and Map strain types that may be responsible for pathogenesis and host specificity; identify strain-specific genes and further our understanding of the evolutionary relationship of Map and the larger Mac complex. This may lead to the identification of specific target regions for earlier and accurate detection and typing of Map as well as improve our understanding of host specific differences and pathogenicity. The knowledge gained may also be used to help improve specific control measures for the Map strain types.

## Results

### Genome content

A total of 268 Map genomes were analysed in this study comprising 205 Type C, 52 Type S, and 4 Type B Map isolates as well as seven isolates from the *M. avium* complex (Mac). Of the 268 isolates there are 243 draft genomes and 25 closed genomes;eight of these were isolates from the Australian Johne’s Disease Reference Collection (AJDR) and were comprised of 6 Type C Map, 1 Type S Map and 1 Type B Map and were generated in this study using both long and short read sequences (Additional file 1). The average genome size of all the annotated Map genomes was approximately 4.7 Mbp in length (ranging from 4,651,437 to 4,959,187 bp) and an average GC content of 69.31% (ranging from 69.24% to 69.37%). The average genome size of all the annotated Mac genomes excluding Map was approximately 5.2 Mbp in length, ranging from 4,953,610 to 5,511,579 bp (Additional file 1). Each Map genome examined in this study contained 3 rRNA genes, 1 tmRNA gene and between 53 to 63 copies of the tRNA gene.

A total of 6,053 genes were identified in the 261 Map genomes, of which 4,108 genes (68%) were present in all strains (the core genome) and 1,945 (32%) were accessory genes (Table 1). Pan-genome analysis revealed variations in the accessory genomes between the three strain types of Map. The Type S pan-genome contained 5,046 genes, with 4,392 (87%) being core genes and 654 (13%) accessory genes. The Type C pan-genome consisted of 5,364 genes, with 4,274 (80%) being core genes and 1,090 (20%) accessory genes and the Type B pan-genome contained 4,798 genes, with 4,298 (89%) being core genes and 500 (11%) accessory genes. Average nucleotide identity (ANI) was calculated between pairs of all genome sequences analysed in this study (data not shown) to determine the sequence similarity between the Map strain types. All type C and B strains shared an ANI of 99.9% and Type S strains shared an ANI of 99.8%.

**Table 1:** Pan-genome statistics of *M. avium* Complex (Mac), *M. avium* subsp. *hominissuis* (Mah), *M. avium* subsp. *avium* (Maa), *M. avium* subsp. *paratuberculosis* (Map), Type C Map, Type B Map and Type S Map

	**Mac**^**a**^	**Mah**^**b**^	**Maa**^**c**^	**Map**^**d**^	**Type S**^**e**^	**Type C**^**f**^	**Type B**^**g**^
**Core genes**	1,899	4,253	4,130	4,108	4,392	4,274	4,298
**Accessory genes**	8,521	1,494	948	1,945	654	1,090	500
**Total genes**	10,420	5,747	5,078	6,053	5,046	5,364	4,798

^a^Mac (*n* = 7)

^b^Mah (*n* = 2)

^c^Maa (*n* = 2)

^d^Map (*n* = 261)

^e^Type S Map (*n* = 52)

^f^Type C Map (*n* = 205)

^g^Type B Map (*n* = 4)

**Fig. 1:**
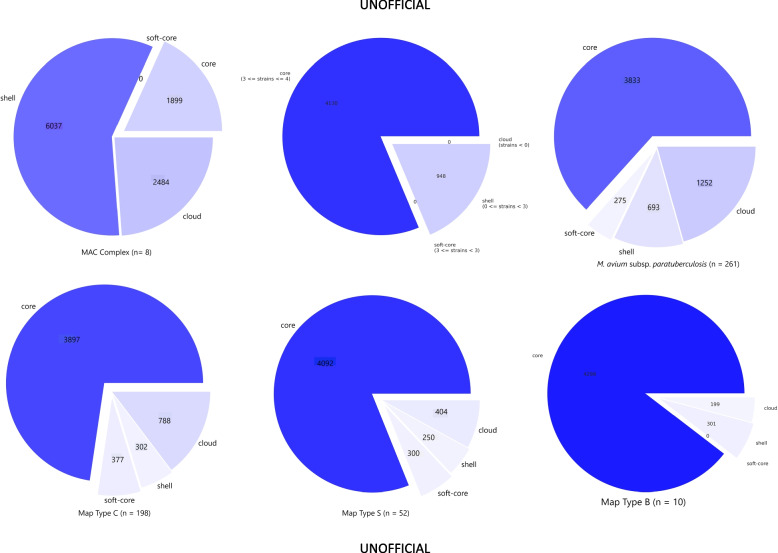
Pie plots describing the pan-genome of Mac, Maa, Map, Map Type S, Map Type C and Map Type B. The core genome is defined as genes present in 99–100% of strains, soft core 95–99%, shell 15–95% and cloud 0–15%. The number of genomes in each pan genome is indicated as ‘n’

Pan-genome pie charts of Mac, Maa, and Map (Fig. 1) describe the core, soft core, shell and cloud genome content and show that Mac has a very large number of accessory genes, 8,521 and only 1,899 core genes while Maa has 4,130 core genes and only 948 accessory genes.

**Fig. 2:**
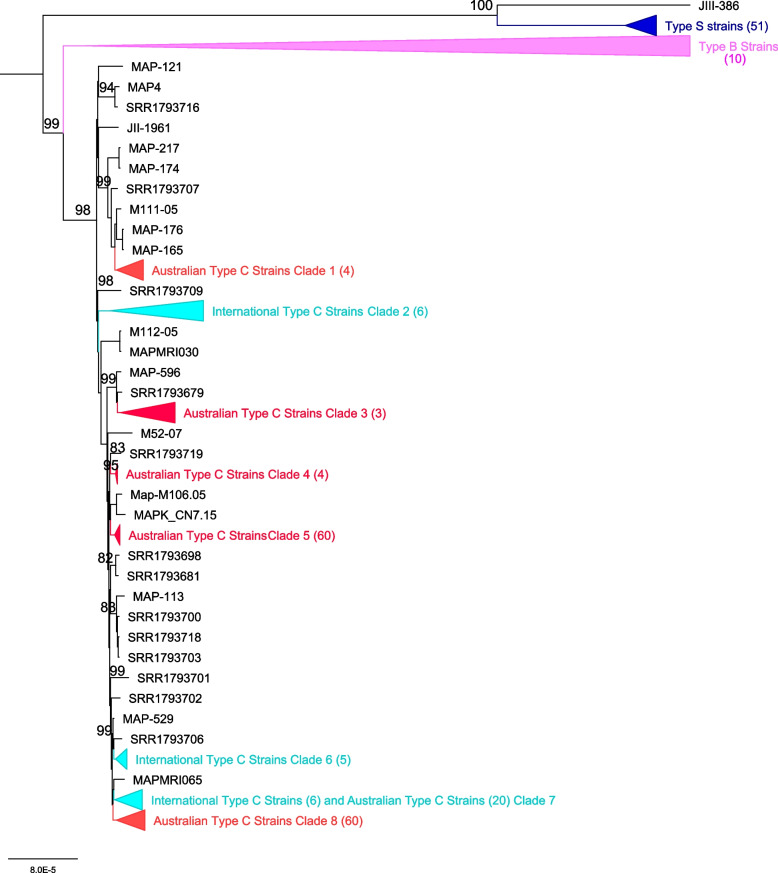
Phylogeny of Australian and international Map genomes based on core genome alignments generated by Roary. Branches show bootstrap support and have been collapsed into clades for ease of presentation

Phylogenetic analysis of the core genome of all Map isolates identified two distinct lineages, Type S and Type C (Fig. 2). The 50 Australian Type S strains clustered separately from the international Type S strain JIII-386 which was consistent with previous core SNP phylogeny where all 58 Australian Type S strains clustered separately to the nine international Type S strains [6]. The majority of the 205 Australian Type C strains clustered together and were distinct from the international Type C strains except for 20 Australian Type C isolates that clustered with six international strains in Clade 7 (Additional File 1). A further eight Australian Type C strains did not group into any cluster. Type B isolates clustered together as a separate clade within the Type C strain grouping.

**Fig. 3:**
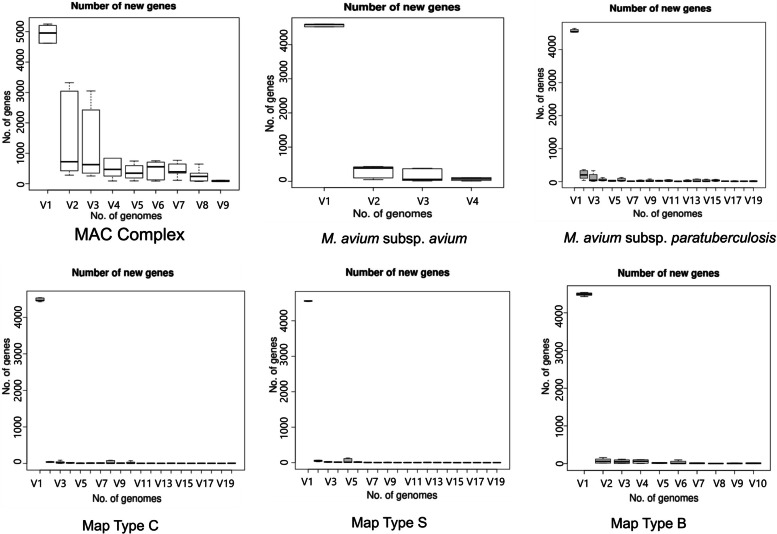
Gene discovery graphs for Mac Complex, *M. avium* subsp. *avium*, *M. avium* subsp. *paratuberculosis*, Map Type S, Map Type C and Map Type B demonstrating the number of new genes that will be added to the pan-genome with the addition of more genomes. For ease of presentation 20 genomes were selected to represent Map, Map Type S and Map Type C graphs

**Fig. 4:**
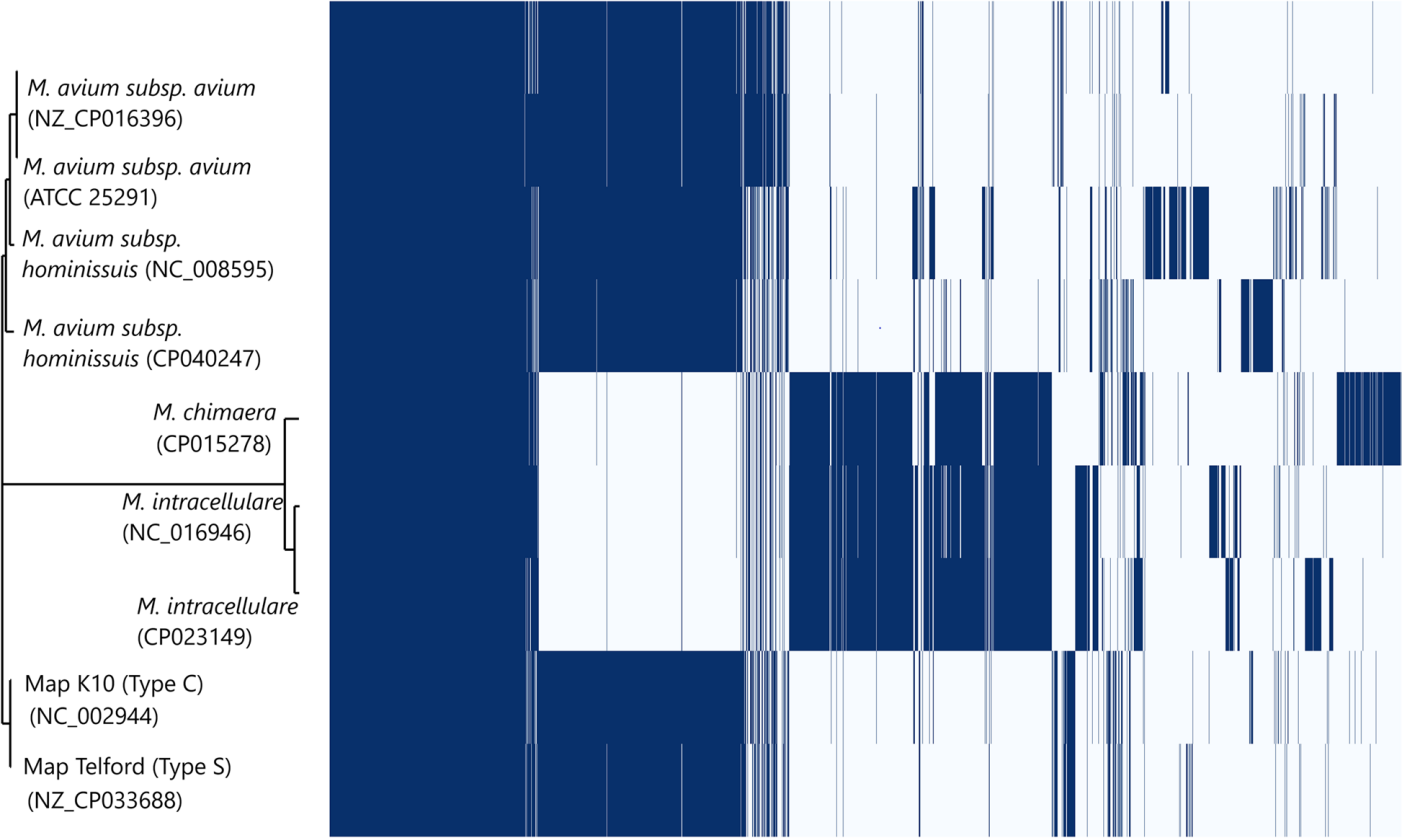
Cluster matrix of 6 species of the Mac complex genomes with dendogram based on homology presence (dark blue) and absence (light blue). Species groupings are determined by core gene phylogeny

**Fig. 5:**
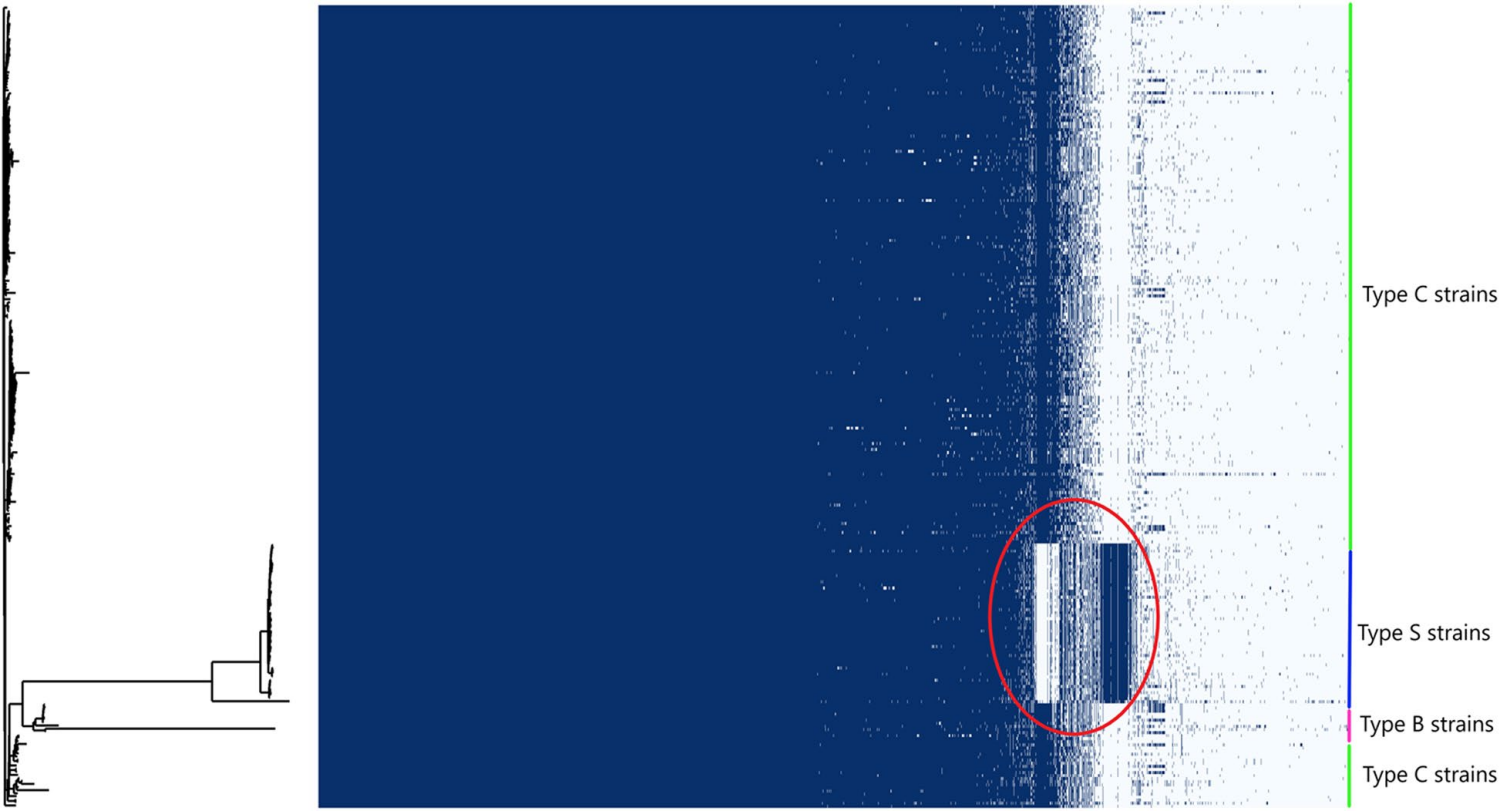
Cluster matrix of the 261 Map genomes analysed in this study with dendogram based on homology presence (dark blue) and absence (light blue). Strain types are indicated by a coloured line and determined by core gene phylogeny. The red circle represents unique homologous regions in Map Type S genomes

Gene discovery graphs for Mac, Maa and Map, and the three strain types of Map (Type S, Type C and Type B) indicate that the number of new genes identified within a genome approaches zero with the addition of each further genome for Map; for example for all Map strains after 17 genomes with a total of 261 genomes analysed the number of genes went from 6,053 to 0, for Type S after 6 genomes with a total of 52 genomes analysed the number of genes went from 5,046 to 0, for Type C after 11 genomes with a total of 198 genomes analysed went from 5,364 to 0 and for Type B after 7 genomes with a total of 10 genomes analysed went from 4,798 to 0 (Fig. 3). Analysis of the homologue cluster matrix clearly grouped the Map separately to the other Mac species and the Map genomes grouped into two distinct lineages, Type S and Type C (Fig. 4). However, five Map isolates (DT3, MAPMRI0103, MAPK_JJ1/13, MAPK_CN4/13 and MAPK_JB16/15) were typed differently by the core gene phylogeny in comparison to previous core SNP phylogeny [6]. DT3 and MAPMRI0103 were previously aligned with Type C strains by core SNP phylogeny but clustered more closely with Type B and Type S strains respectively in the core gene phylogeny. Isolate DT3 however was significantly genetically divergent from the other bison strains, and this was taken into consideration when performing further downstream analysis. All Type S and Type C strains clustered into two distinct lineages with visible unique homologues between the two Map strains (Fig. 5). The core gene phylogeny also demonstrates the close genetic relatedness of the Type C and Type B strains of Map.. Three international isolates (MAPK_JJ1/13, MAPK_CN4/13 and MAPK_JB16/15) from South Korea that were previously strain typed as Type C [19] were strain typed as Type B in this study using core SNP phylogenetic analysis (data not shown) and core gene phylogeny.

### Structural comparative analysis of Mac and Map genomes

**Fig. 6:**
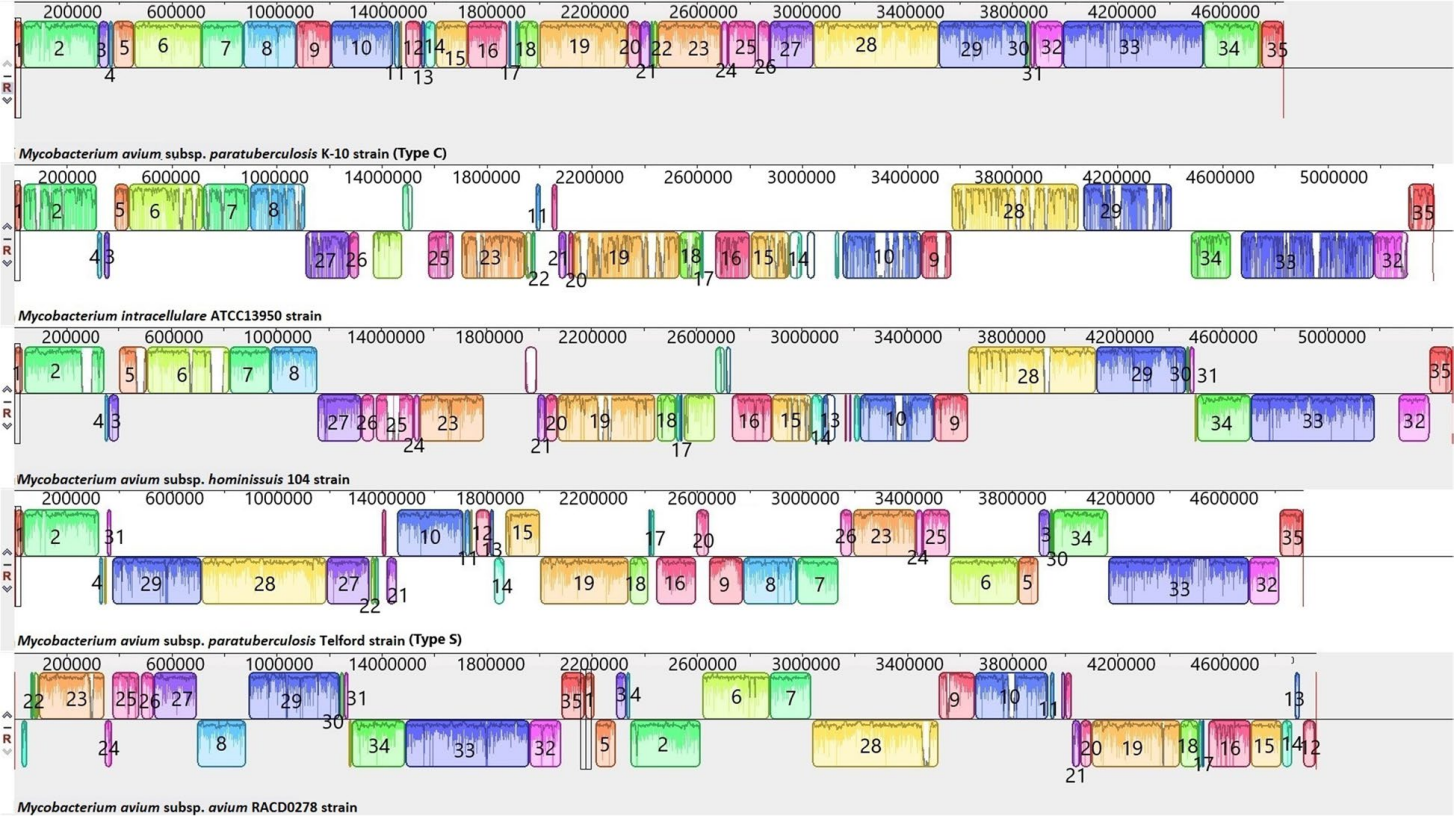
Pairwise comparison of M. hominissuis, M. avium subsp. avium, M. intracellulare, Telford Type S Map and K-10 Type C Map, genome sequences using Mauve. Homologous segments among the strains are represented by identically coloured boxes and assigned the same number

**Fig. 7:**
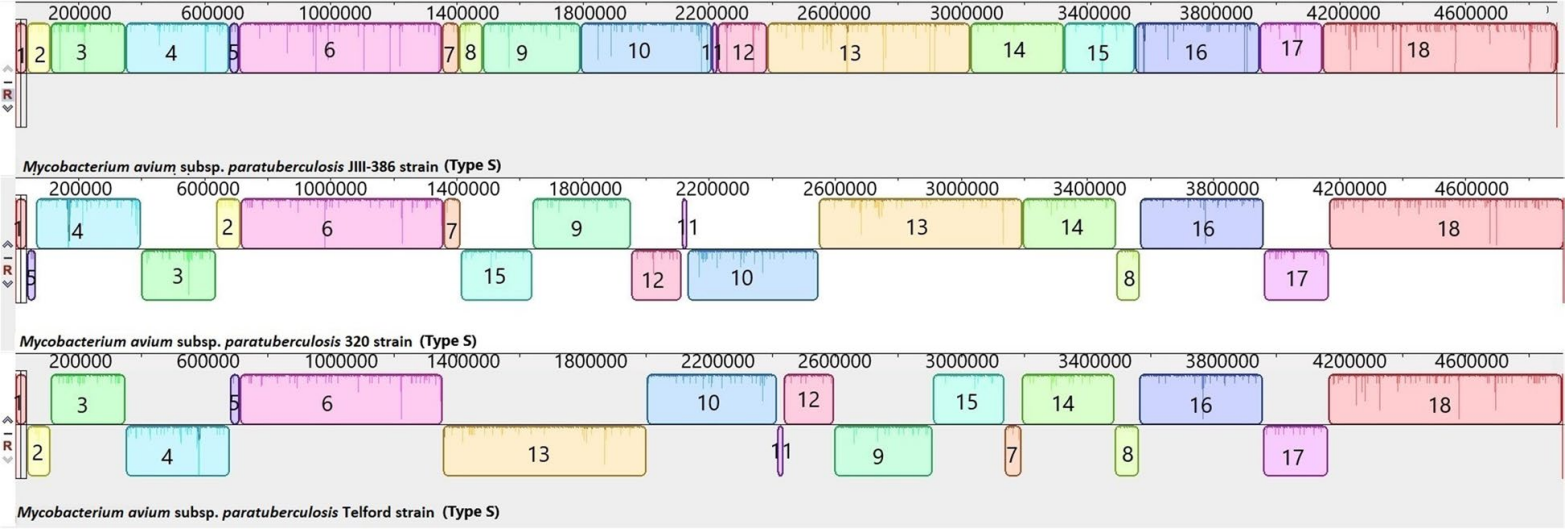
Pairwise comparison of three Type S genome sequences using Mauve. Homologous segments among the strains are represented by identically coloured boxes and assigned the same number

**Fig. 8:**
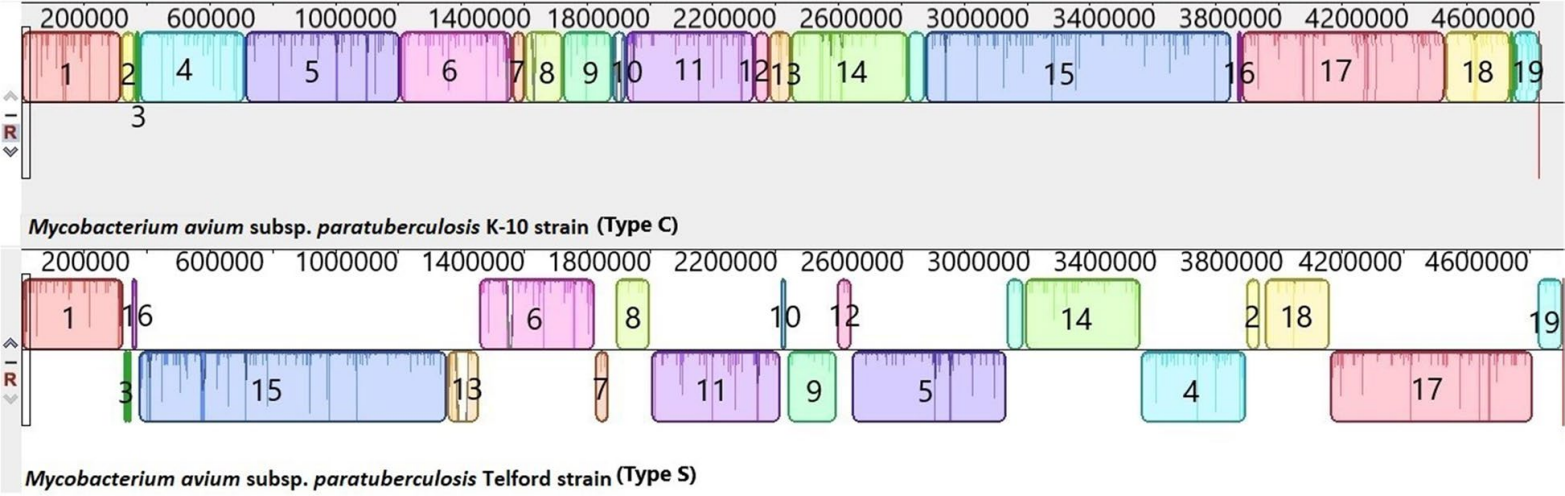
Pairwise comparison of Telford Type S Map and K-10 Type C Map. Homologous segments among the strains are represented by identically coloured boxes

**Fig. 9:**
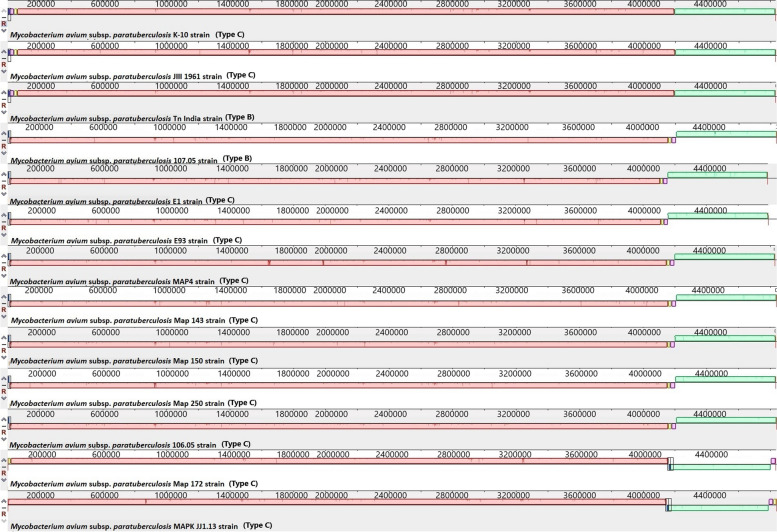
Pairwise comparison of 11 Type C and 2 Type B genomes using Mauve. Homologous segments among the strains are represented by identically coloured boxes

The complete closed genomes of the Map Type C K-10 isolate, the Map Type S Telford isolate, Maa, Mah and *M. intracellulare* were aligned to identify structural rearrangements between Map and Mac. A large homologous region approximately 11,000 bp in length was present in both the *M. intracellulare* and Mah genomes that was not present in the genome of either Maa or the S or C strains of Map. The genome of *M. intracellulare* contains five small regions spanning ~ 140 to 160kb, ~ 210 to 215kb, and ~ 300 to 315kb which have no homology to regions in the other Mac species nor in the Map strains. Similarly, Maa (Accession: NZ_CP016396) contains four small regions spanning ~ 100kb to 105kb, 1.3Mb to 1.32Mb and 3.9Mb to 4.01Mb and Mah has three regions ~ 190kb to 200kb, ~ 250kb to 270kb that have no homology to regions in the other Mac or Map isolates. These genomic regions are mostly IS elements, hypothetical proteins, and some metal transporter proteins. The Telford (Accession: NZ_CPO33688.1) Type S Map genome has two unique regions at position 380kb and 1.4Mb compared to the other Mac and the K-10 Type C Map genome (Fig. 6) while the Map the K-10 (Accession: NC_002944.2) Type C Map genome only has one unique region at position 4.73Mb compared to the other Mac and Type S genomes. The alignments of the 19 complete Type C, two Type B and three type S genomes revealed that the Type C and Type B strains are highly conserved structurally, while more rearrangement and diversity was observed within the Type S strains when compared to the Type C and the Type B strains (Figs. 7, 8 and 9). The three Type S strain genomes (Telford, JIII-386 and Map-320) showed 11 inversions and 12 rearrangements when compared to each other (Fig. 7). There were nine inversions and 10 rearrangements between the Type S Telford reference genome and the Type C K-10 reference genome (Fig. 8).

**Fig. 10:**
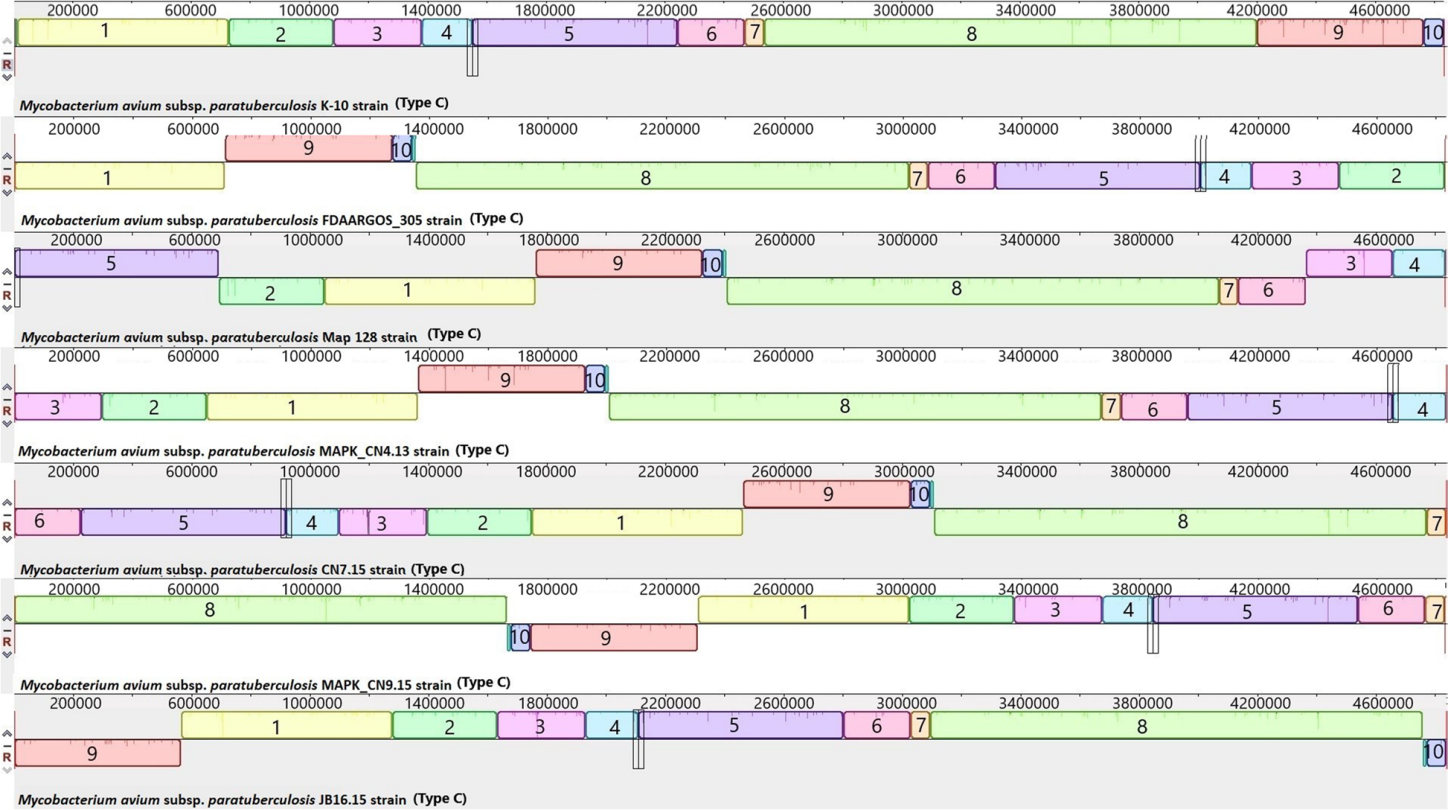
Pairwise comparison of 7 genome sequences (Mycobacterium avium K-10 strain Accession: NC_002944.2, FDAARGOS_305 Accession: NZ_CP018019.1, Mycobacterium 128 strain Type C field isolate, Mycobacterium MAPK_CN4.13 strain Sequence ID: CP033910.1, Mycobacterium MAPK_CN7.15 Sequence ID: CP033428.1, Mycobacterium MAPK_CN9.15 strain Sequence ID: CP033427.1, and Mycobacterium MAPK_JB16.15 Sequence ID: CP033911.1) using Mauve showing the nine inversions and one rearrangement compared to Map K-10. Homologous segments among the strains are represented by identically coloured boxes and assigned the same number

The genomes of 13 Type C strains including the K-10 reference genome were structurally identical (e.g. there were no rearrangements or horizontal gene transfer evident amongst these strains) (Fig. 9). The remaining six Type C genomes were structurally alike, however there were nine inversions amongst these genomes and one rearrangement observed in the K-10 genome (Fig. 10). The Type B Tn-India genome and the Type C JIII-1961 genome were structurally identical to each other and differed from the K-10 Type C genome with a reverse complimentary orientation of their genome at position 4.1Mb to 4.2Mb (Fig. 9).

**Fig. 11:**
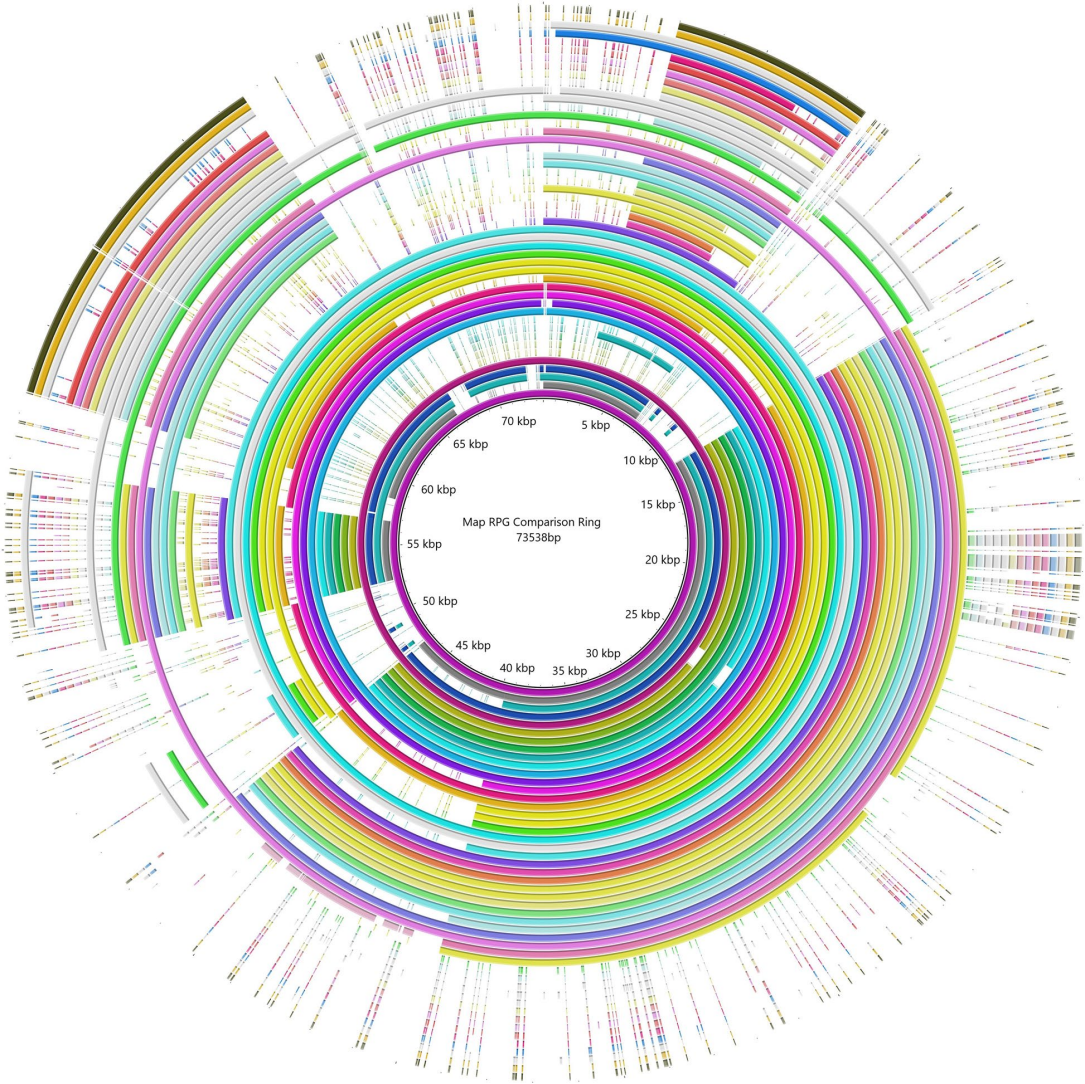
Comparison of regions of genome plasticity of 48 selected Map genomes were determined by Ppanggolin. The reference genome K-10 is represented in the inner purple ring. The first 31 inner rings are Type C Map, and the 14 most outer rings are Type S Map

Comparison of Type C K-10 reference genome with the *M. avium* subsp. *avium* genome (accession NZ_CP016396) using blastp revealed 13 regions of genome plasticity that were absent from the *M. avium* subsp. *avium* genome (Additional file 2). There were four regions of genome plasticity in the Type S genome that were not present in Type C or Type B genomes with the largest region of genome plasticity, consisting of 15.4kb, that was present in the Type S Telford genome (accession NZ_CPO33688.1) but absent from the Type C K-10 genome (accession NC_002944.2). A total of 1,493 regions of genome plasticity was identified in Map (Fig. 11). Analysis of the genes present within these regions of genome plasticity identified 27 genes that were present in Type S genomes but absent from both Type C and Type B, and comparison of the presence/absence data from Roary identified an additional 22 Type S strain genes that were not present in Type C and Type B strains (Additional file 2). Overall, the analysis of the genome content of Map has identified 87 core genes that are present in all the Map strain types but absent from the four Mac genomes analysed, and a further 49 core genes that are unique to Map Type S. There were no unique core genes present in the Type C and Type B strains that were not present in Type S Map. All the unique core genes identified in Map and Type S were also mapped back to the reference genomes (Map K-10, *M. avium* subsp. *hominissuis*, *M. avium* subsp. *avium*, *M. avium* subsp. *intracellulare*, and *M. chimaera*) and it was found that the 87 genes present in Map did not map back to the four Mac reference strains and the 50 unique genes identified in Type S Map did not map back to the Type C reference strains using Geneious Prime.

### Effector delivery systems and effector proteins in Mac and Map

Genes associated with effector delivery systems and effector proteins, as defined by the Mycobacterial virulence database (http://www.mgc.ac.cn/cgi-bin/VFs/genus.cgi?Genus=Mycobacterium), were identified in the 268 genomes analysed and presence and absence of these genes between Mac and the Map strain types was compared (Table 2). Five out of the eight genes associated with the ESX-2 delivery system were present in all the Mac and Map genomes except for PPE69 which was absent from *M. intracellulare*. There were only six of nine genes associated with the ESX-3 effector delivery system present in the Mac and Map genomes of which eccB3 was not present in Maa and Mah, espG3 was not present in *M. intracellulare* and PPE4 was only present in two (MAP-594 and JIII-386) out of the 52 Type S genomes and was absent from *M. intracellulare*. Only two out of the four ESX-4 associated genes were present in the Mac and Map genomes, but none of the genes were present in *M. intracellulare*. Eight out of the 12 ESX-5 associated genes were present in Map and Mac except for mycP5 gene which was absent from *M. intracellulare*. One out of the two genes associated with the ESAT-6 effector delivery system was present in all the Mac and Map genomes. None of the 22 genes associated with the ESX-1 effector delivery system were identified in any of the Mac species or Map. There was no difference in the presence and absence of effector proteins between Type C and Type B Map strains except for PPE4 which was present in only 2 of the 52 Type S strains analysed.

**Table 2:** Effector delivery systems and their related genes in the Mac species and Map strains identified by the Mycobacterial virulence database

Genes	Effector delivery system	Mah^a^	Maa^b^	*M. Intracellulare*	Type S Map	Type C Map	Type B Map
caeA	carboxylesterase	+	+	+	+	+	+
eccA1	ESX-1	-	-	-	-	-	-
eccB1	ESX-1	-	-	-	-	-	-
eccCa1	ESX-1	-	-	-	-	-	-
eccCb1	ESX-1	-	-	-	-	-	-
PE35	ESX-1	-	-	-	-	-	-
eccD1	ESX-1	-	-	-	-	-	-
espK	ESX-1	-	-	-	-	-	-
eccE1	ESX-1	-	-	-	-	-	-
mycP1	ESX-1	-	-	-	-	-	-
espD	ESX-1	-	-	-	-	-	-
espC	ESX-1	-	-	-	-	-	-
espA	ESX-1	-	-	-	-	-	-
espB	ESX-1	-	-	-	-	-	-
PPE68	ESX-1	-	-	-	-	-	-
Espl	ESX-1	-	-	-	-	-	-
espJ	ESX-1	-	-	-	-	-	-
espL	ESX-1	-	-	-	-	-	-
espR	ESX-1	-	-	-	-	-	-
espE	ESX-1	-	-	-	-	-	-
espF	ESX-1	-	-	-	-	-	-
espG1	ESX-1	-	-	-	-	-	-
espH	ESX-1	-	-	-	-	-	-
eccC2	ESX-2	+	+	+	+	+	+
eccD2	ESX-2	+	+	+	+	+	+
espG2	ESX-2	+	+	+	+	+	+
esxC	ESX-2		-	-	-	-	-
esxD	ESX-2		-	-	-	-	-
mycP2	ESX-2	+	+	+	+	+	+
PE36	ESX-2	-	-	-	-	-	-
PPE69	ESX-2	+	+	-	+	+	+
eccA3	ESX-3	+	+	+	+	+	+
eccB3	ESX-3	-	-	+	+	+	+
eccC3	ESX-3	+	+	+	+	+	+
eccD3	ESX-3	+	+	+	+	+	+
espG3	ESX-3	+	+	-	+	+	+
esxG	ESX-3	-	-	-	-	-	-
esxH	ESX-3	-	-	-	-	-	-
PE5	ESX-3	-	-	-	-	-	-
PPE4	ESX-3	+	+	-	Present in 2/52	+	+
eccB4	ESX-4	+	+	-	+	+	+
esxT	ESX-4	-	-	-	-	-	-
esxU	ESX-4	-	-	-	-	-	-
mycP4	ESX-4	+	+	-	+	+	+
eccA5	ESX-5	+	+	+	+	+	+
eccB5	ESX-5	+	+	+	+	+	+
eccCb5	ESX-5	+	+	+	+	+	+
eccD5	ESX-5	+	+	+	+	+	+
eccE5	ESX-5	+	+	+	+	+	+
esxM	ESX-5	-	-	-	-	-	-
esxn	ESX-5	-	-	-	-	-	-
mycP5	ESX-5	+	+	-	+	+	+
PE18	ESX-5	-	-	-	-	+	-
PE19	ESX-5	-	-	-	-	-	-
PPE25	ESX-5	+	+	-	+	+	+
PPE26	ESX-5	+	+	-	+	+	+
eccA2	ESAT-6	+	+	+	+	+	+
espR	ESAT-6	-	-	-	-	-	-
MAP0163	Hypothetical protein	+	+	+	-	+	+
MAP1504	Hypothetical protein	-	-	-	-	-	-
MAP1509	Hypothetical protein	+	+	+	+	+	+
MAP4242	Hypothetical protein	+	+	-	+	+	+

^a^M. avium subsp. hominissuis

^b^M. avium subsp. avium

Three proline-glutamate (PE) genes were found in all Map strains and 27–29 PPE genes were found in Type C and Type B strains and 30–32 PPE genes in Type S strains. PPE2 gene was not present in any of the Type C genomes, and PPE15 and PPE18 were not present in any of the Type S genomes. The largest subfamily of PE family genes, PE-PGRS, had 2 copies in Type S and C and 4 copies in Type B. This gene family also had two copies in Maa and *M. intracellulare*, and four copies in Mah and M. *chimaera*.

Twenty of the 60 effector proteins that have been identified from other Map-related studies (Additional file 3) were present in all Mac and Map genomes. There were only 20 effector proteins present in the *M. intracellulare* genome and this was the least amongst the Mac species; with 34, 36, 33 and 33 effector proteins present in Maa, Type S, Type C and Type B strains respectively (Table 2). Of the 23 effector proteins that were absent from the Mah genome only five were present in the other Mac species including Map: LSP-12 and MptD was only present in the Map genomes, fldA_4 (Cinnamoyl-CoA:phenyllactate CoA-transferase) was only present in Type S strains, yfnB (aciddehalogenase) and MmpL5 (siderophore exporter) was present in Maa and Type C and Type B strains of Map (Additional file 3). There was no difference in the presence/absence of effector protein genes between Type C and Type B Map. There were 30 effector proteins that were present in all the three Map strain types (Table 2). There were six effector proteins (Phospho-2-dehydro-3-deoxyheptonate aldolase, Cinnamoyl-CoA:phenyllactate CoA-transferase, 7-beta-hydroxysteroid dehydrogenase (NADP( +)), Putative short-chain type dehydrogenase, Diacylglycerol kinase and Formyl-CoA:oxalate CoA-transferase) that were present in Type S but absent from both Type C and Type B Map strains; and only three effector proteins (2-haloalkanoic acid dehalogenase, Siderophore exporter MmpL5, and a polyketide synthase-associated protein family involved in cell adhesion) that were present in Type C and Type B Map but absent from Type S (Additional file 3).

### Functional categories and classes of the Mac and Map pan-genome proteins

**Fig. 12:**
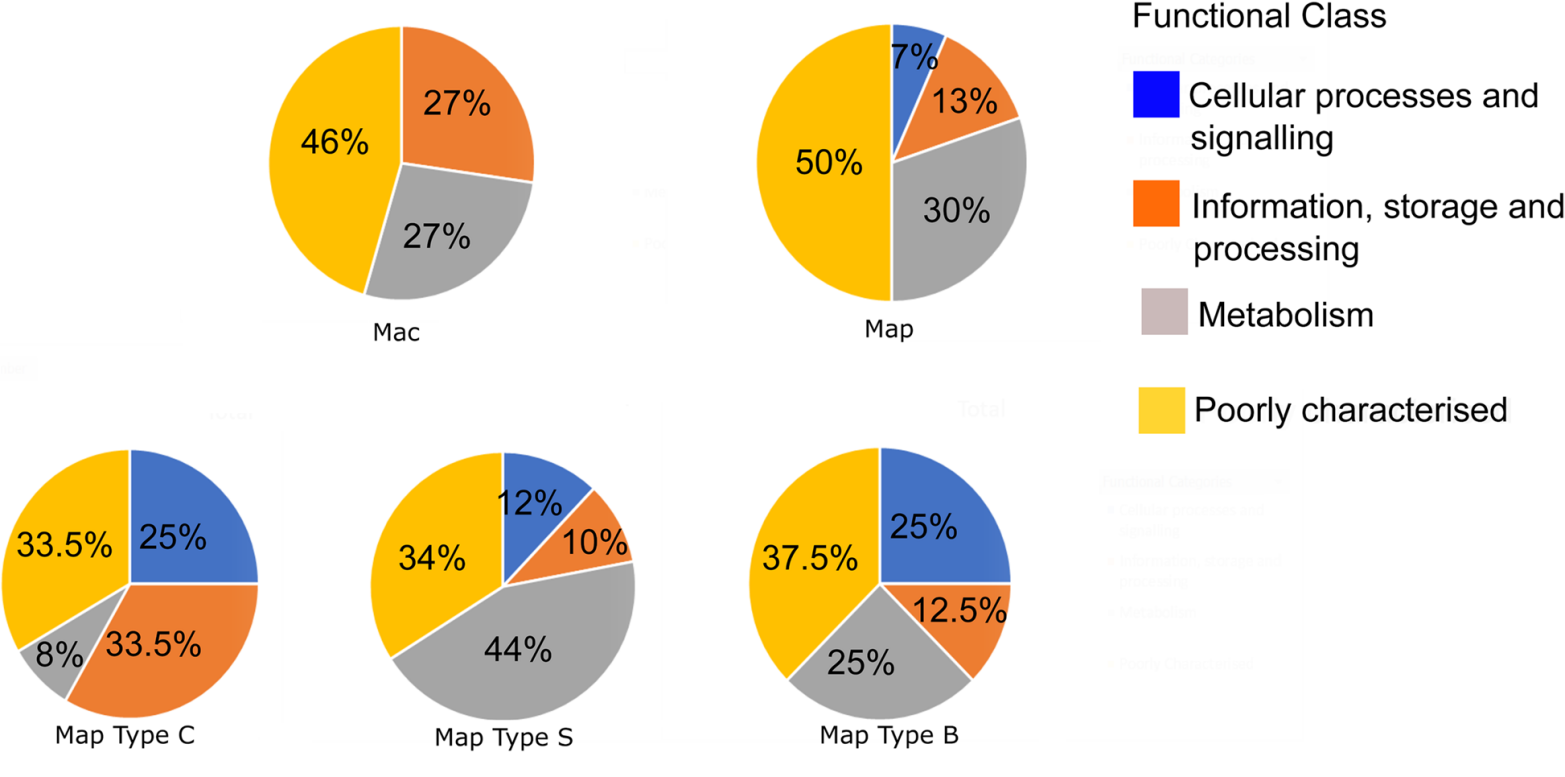
Functional categories of proteins in the Mac, Map, Map Type C, Map Type S and Map Type B genomes. The functional categories are grouped into four classes and are indicated by colour as described in the legend

The functional categories that were assigned to the pan-genome proteins were grouped into four functional classes: Cellular processes and signalling; Information, storage and processing; Metabolism and “Poorly characterised” which included proteins that did not have an ortholog match in the eggNOG database. Almost half of the proteins remain poorly characterised for Mac and Map (46% and 50% respectively). Of the remaining characterised proteins for Mac and Map “metabolism” was the most abundant functional category (30% and 27% respectively), followed by “information, storage, and processing” (13% and 27% respectively) and “cellular processing and signalling” (7% and 0% respectively) (Fig. 12).

Within the different Map strain types there were some significant differences between the protein classes. There was a higher incidence of “information, storage and processing” proteins in Type C strains (33.5%) compared to Type S (10%); the metabolism related proteins was higher in Type S (44%) compared to Type C and Type B (8% and 25% respectively); cellular processing and signalling related of proteins were higher in both Type C and Type B (25%) compared to Type S strains (12%); while poorly characterised proteins were similar between the 3 strain types (Fig. 12).

### Carbohydrate active enzyme profiles (CAZymes)

There were some differences in the CAZyme family profiles between Mac and Map. For example, *M. chimaera* and *M. intracellulare* did not contain any copies of the CBM2 CAZyme family whilst Mah, Maa and Map all contained one copy. *M. chimaera* and Mah had eight and nine copies of the CE5 CAZyme family respectively which is lower than the eleven copies found in Map, *M. intracellulare* and *Maa*. There was also a notable difference with the copy number of the CE10 Cazyme family between the Mac and Map genomes with Map having 14 to 17 copies, *M. chimaera* having 20 copies of CE10 and *M. intracellulare* and *Mah* having 18 and19 copies respectively.

Eight CAZyme families were identified in all Map genomes: CBM2, CBM48, CE1, CE10, CE14, CE4, CE5 and CE9 (Additional file 8). The copy number of CAZyme families present in each strain type did not vary greatly, with one copy of CBM2, CMB48 and CE9 in all strain types, 2–3 copies of CE14, three copies of CE4 and 10–11 copies of CE1 and CE5 present in all strains ( Additional file 9). The most notable difference in the CAZyme profile was with the CE10 CAZyme family where there were 17 copies in 193 of the Type C strains and all four Type B strains and 14 Type S strains, while the remaining 49 Type S strains contained only 16 copies of the CE10 CAZyme family.

### Secondary metabolite gene clusters

Analysis of the 25 closed Mac and Map genomes using antiSMASH 5.1.2 identified eight genomes (Three Type C, two Type S, one Type B, *M. hominissuis* and *M. avium avium*) that contained 16 secondary metabolite clusters, 15 genomes (14 Type C and one Type B) that contained 17 secondary metabolite clusters and one Type S genome (JIII-386) that contained 14 secondary metabolite clusters. The *M. intracellulare* closed genome contained 20 secondary metabolite clusters which is the highest amongst the Mac and Map closed genomes analysed in this study (Additional file 7). All the Mac and Map isolates contained a terpene metabolite cluster with 71% identity to the antiSMASH database, a non-ribosomal peptide synthetase cluster (NRPS) with the predicted metabolite synthesis function for mycobactin with 80–90% similarity to the database, a mixed T3PKS.T1PKS cluster with the predicted metabolite synthesis function for methylated alkyl-resorcinol/methylated acyl-phloroglucinol and a T3PKS polyketide metabolite cluster with the predicted metabolite synthesis function for alkyl-resorcinol with 100% similarity to the database. The T3PKS polyketide metabolite cluster was not present in the JIII-386 Type S isolate. Ten of the metabolite clusters all had very low similarities to the antiSMASH database (6–35%), including 4 NRPS, 2 T1PKS, 2 NRPS.T1PKS, 1 T1PKS.NRPS and 1 T1PKS with a predicted herbimycin A metabolite synthesis function. There were either one or two NRPS-like metabolite clusters present in all the genomes that had no identity to the database.

All the Map genomes except for Type S strain JIII-386 contained a secondary metabolite NRPS cluster with the predicted function for chloromyxamide synthase activity. This secondary metabolite gene cluster was not present in the Mac genomes analysed in this study. All the Map Type C and Type B genomes possessed 5 PKS, 5 NRPS and 2 mixed NRPS.PKS clusters, whereas the Type S genomes only harboured 4 PKS (except for the Telford strain which had 5), 4 NRPS and 3 mixed NRPS.PKS clusters. All Type S strains, as well as the Type C K-10 isolate, did not contain the metabolite cluster encoding the type 1 polyketide synthase cluster (T1PKS, predicted product vazabitide A) which is a biosynthetic gene cluster from *Streptomyces sp*. The significance of this is unknown as the presence of this gene cluster in the Type C genomes (excluding K-10) and Type B genomes only had a very low similarity of 8% identity to the database.

### Insertion sequences

IS Finder did not identify any IS elements in the 243 draft genomes analysed in this study. However, in the 25 closed genomes ten different IS elements were identified from four different IS families (IS110, IS256, IS481, and IS1182) (Additional file 5). *M. hominissuis* contained the highest number of different IS elements in the genome within Mac with 17 IS elements from five different IS families. *M. avium avium* contained only nine different IS elements from four different IS families and *M. intracellulare* contained only one IS element belonging to the IS3 family (Additional file 6). All the Map strains contained the same 10 IS elements from four different IS families except for the Type B strain Tn-India which did not have the IS element ISmgi2. The number of copies of an IS element identified in these genomes differed between the Map strain types. For example, the Type S genomes had 22 copies of IS900 (IS110 family) and nine copies of IS1245 (IS256 family) insertion element whereas Type C and Type B had between 17–19 copies of the IS900 and between five to eight copies of the IS1245.

### Integrated prophages and prophage-like elements

In the Mac complex there were two incomplete prophage regions identified in *M. avium* subsp. *avium*, two incomplete and one intact prophage region in *M. chimaera*, one incomplete prophage region in *M. intracellulare* and four incomplete, one questionable and one intact prophage region in *M. hominissuis*. There was one questionable and eight incomplete prophage and prophage-like regions identified in Type C Map isolates, of which two of these regions were identified as *Rhodococcus* phage REQ2 and Streptomyces phage. In Type B Map strains there were three incomplete prophage and phage-like regions, two of which were also identified as *Rhodococcus* phage REQ2 and the Streptomyces phage that was identified in Type C Map (Additional file 4). In Type S Map strains there was one questionable and six incomplete prophage and prophage-like regions, one of which was identified as Mycobacterium phage Che.

### Plasmids

Of the Mac genomes analysed in this study, one unnamed plasmid was identified in *M. chimaera.* There were no plasmid DNA sequences identified from any of the 261 Map genomes analysed.

### CRISPR

CRISPRCasFinder was used to identify the CRISPR elements and cas related genes present in the seven Mac and 261 Map genomes. A total of 45 unique but hypothetical CRISPR elements were identified in the 268 Map and the 5 Mac genomes analysed in this study (Additional file 10). However, there were no cas genes identified in any of the Map or Mac genomes. *M. avium, M. intracellular* and *M. hominissuis* and *M. chimaera* had five, seven, eight and eight CRISPR elements respectively. *M. avium*, *M. hominissuis* and Map shared one identical CRISPR element. The CRISPR elements identified in both *M. intracellular* and *M. chimaera* were not present in any of the other Mac or Map genomes analysed.

There was no CRISPR element that was common to all Map strains. All Type S strains contained three CRISPR elements except for the JIII-386 isolate which contained 7 CRISPR sequences. There was one CRISPR element (> cpr04_4, Additional file 10) that was present in all the Type S strains and in 102 of the 205 Type C strains and three of the four type B strains (Additional file 11). In the Type C strains the number of CRISPR elements present in the genomes ranged from six to ten, with seven of the CRISPR elements identified in 84 of the genomes while 10 CRISPR elements were only identified in one genome. There were common CRISPR elements amongst the Type C genomes, but there was not one unique CRISPR element in all the Type C strains. The number of CRISPR elements in the four Type B strains ranged from six to eight; like the Type C strains there was not one unique CRISPR element present in all the Type B genomes.

## Discussion

This study represents the largest comparative genomic study of Mac and Map type strains to date with 263 draft genomes and eight closed genome sequences generated for analysis. The average length of the Map genomes was 4.8Mb long, had a GC content of 69.3% and the number of tRNAs ranged from 53 to 63, which is in contrast to previous reports that have reported 46 [14] and 45 [10] tRNAs. The Map pan-genome contained 4108 core genes and 1945 accessory genes while the Mah and Maa pan-genomes contained 4253 core genes and 1494 accessory genes, and 4130 core genes and 948 accessory genes, respectively.

*Mycobacterium avium subsp. hominissuis* (Mah) is a pathogen of humans and pigs [21] whereas *Mycobacterium avium* subsp. *avium* (Maa) is an avian pathogen, mainly restricted to avian species. Maa has also been reported on rare occasions to have been isolated from pigs, cattle, goats, dogs, cats, horses and some other wildlife species [22]. Map, on the other hand, is a pathogen of ruminants, mainly cattle and sheep but has also diversified successfully into other animal species especially those that interact closely with ruminants such as rabbits, pigs, dogs and horses [23]. The adaptation of Map to this diverse host range may be attributed to the larger accessory genome found in Map compared to Mah and Maa. Similarly, Type C Map strains had the largest accessory genome accounting for 20% of their pan-genome, compared to 13% in Type S strains. The larger accessory genome of Type C Map may be due to its more extensive host range and genomic plasticity when compared to Type S Map. The gene discovery graphs indicated that Map has a closed genomes as defined by Medini et al. [24]. Other examples of closed pan-genomes are *B. anthracis*, *M. bovis* and *Chlamydia trachomatis*, with an extreme example being *Bucknera aphidicola* whose genome has undergone no changes in the last 50 million years [25]. Map, Type S, Type C and Type B strains reached saturation after sequencing 17, 6, 11 and 7 genomes respectively. The Mac genomes reached saturation after 9 genomes and Maa approached 0 after 4 genomes, however more Mac and Maa genomes should be includedto confirm these results (no more Mac and Maa genomes were available at the time of this study).

Phylogenetic SNP analysis from a previous study [6], as well as the phylogenetic core genome analysis (Fig. 1) and the homologue matrix (Fig. 5) conducted in this study support the two lineages within Map; Type S and Type C. The homologue matrix has highlighted that there is a large region of unique genes in Type S strains that are not present in Type C strains and has also shown that there is very little genetic variation between Type C and Type B strains (Fig. 5). This finding was confirmed by the PPanGGolin output (Fig. 11) that was viewed using BRIG and ACT and helped to identify 50 unique genes in Type S strains of Map. The homologue matrix showed that the Australian Type C isolates were not significantly genetically different from the international Type C isolates analysed in this study indicating that there is little genetic variation of this strain type distributed across the world.

The mauve alignments of the complete genomes were used to identify rearrangements in the Mac and Map genomes analysed in this study and the results showed that there were numerous rearrangements identified between Mac and Map. This was also evident between Map Type S and Type C strains as there were several rearrangements in Type S Map when compared to Type C Map (Fig. 10). There were 12 rearrangements within the Type S strains themselves compared to only one rearrangement within Type C strains which shows that Type S strains are more heterogenic than Type C strains. This heterogeneity has been previously reported within Type S strains [26, 27] grouping them into two subtypes (I and III) using different molecular typing tools [26]. In contrast Type C and Type B strains show strong synteny with only one structural difference identified between them (a reverse complimentary orientation, Fig. 10).

The differences in functional classes identified between the strain types also reveals the structural differences between the strains especially between Type S and Type C. The Type C isolates had a higher number of genes in the information, storage and processing class and the cellular processing and signalling class. These two functional classes contain cell wall biogenesis and defence mechanism functional categories which may contribute to the success of the Map Type C isolates to survive and infect broader range of animal species.

### Effector delivery systems and effector proteins in Mac and Map

There were no genes in the Map pan-genome associated with the ESX-1 effector delivery system which is consistent with previous findings for Mycobacterial species where the ESX-1 system has been lost in both *M. avium* species and *M. leprae* [28]. The ESX-1 system has been identified to be essential for virulence, however due to its absence in some pathogenic mycobacteria further research into other effector delivery systems identified that ESAT-6 and ESX-5 are involved in virulence [17, 28]. The ESAT-6 delivery system has been identified as being unique to the Actinobacteria [29], which includes Mac and Map and the ESX-5 effector delivery system is specific for pathogenic mycobacteria leading to the hypothesis that it also is involved in virulence [28]. There were 12 genes associated with the ESAT-6 system and two associated with the ESX-5 system identified in the Mac and Map genomes. Almost half of the genes associated with these systems are absent from *M. intracellular* including all the genes associated with the ESX-4 system indicating the more distant evolutionary relationship that *M. intracellulare* has with Mah, Maa and Map.

The PE/PPE gene families play a crucial role in the pathogenesis of Mycobacteria and are believed to be located at the cell surface and involved in immune evasion and linked to virulence [28]. There is a strong selection for the PPE gene family in pathogenic mycobacteria, and both the PE/PPE families comprise from 1% of the Map genome to up to 10% in *M. tuberculosis* [30]. In this study there were 30–32 copies of PPE gene family in Type S strains and 27–29 copies in Type C and Type B strains. The PE-PGRS subfamily of genes, which are involved in the persistence of Mycobacteria and immune evasion and antigenic variation [31], was originally thought to be absent from Map, Maa and Mah [32]. Two subsequent studies did identify one or two homologues of the PE-PGRS gene in the Map genome [14, 31]. Analysis of the larger Mac and Map genome dataset in this study identified four copies of the PE-PGRS gene in Map Type B, *Mah* and *M. chimaera* and two copies in Type C, Type S, Maa and *M. intracellular.* There are four PPE proteins (PPE4, PPE65, PPE25 and PPE26), all members of the ESX-5 and ESX-3 systems that are essential for virulence in Mycobacteria [33]. The PPE4 protein was found in only two out of the 52 Type S strains. The absence of this PPE in Type S strains may account for the difference in their ability to cause disease between cattle and sheep, as Type S strains rarely cause disease in cattle [1]. The presence of PPE proteins in only two of the Type S genomes and how they acquired this effector is unclear.

The mptD effector protein is expressed on the cell surface of Map during infection and is unique to Map as it has never been found in the genomes of other Mac or mycobacterial species [34] which was consistent with the findings in this study. Diacylglycerol kinase (dagK) plays an important role in the biosynthesis of lipids in the cellular membrane of *M. tuberculosis* [35] and contributes to the bacterium’s survival within host cell macrophages and the environment. This effector protein was present in Mah, Maa and Type S Map genomes, but was absent from Type C and Type B Map. The presence of this effector in Type S Map may attribute to the host and cultural characteristic differences between Type C and B Map.

Mycobacteria membrane protein large genes (mmpL) are involved in the biosynthesis of cell wall-associated glycolipids and specifically mmpL5 encodes a protein involved in lipid transport [36]. This study found that the mmpL5 gene was present in all 188 Type C strains and four Type B strains and absent in all 52 Type S strains. This finding is in agreement with the representational difference analysis performed by Marsh and Whittington [37]. Studies have shown that inactivation of the mmpL gene leads to changes in surface characteristics such as altered colony morphology, reduced sliding motility, and reduced biofilm formation. The absence of mmpL5 in Type S strains [38, 39] may indicate that these genes play a role in host association and may also explain the differences in cultural requirements between Type C and Type S strains.

CAZymes are enzymes involved in the breakdown, biosynthesis, or modification of complex carbohydrates and are designated into six classes based on their catalytic function (glycosyltransferases, glycoside hydrolases, polysaccharide lyases, carbohydrate esterases, auxillary activities, and carbohydrate binding modules) [40]. Eight CAZyme families were identified in all the Map genomes in this study, and they belonged to only two of the CAZyme classes; two of the CAZyme families (CBM2 and CBM48) belonged to the carbohydrate binding modules class and six of the CAZyme families belonged to the carbohydrate esterases class. Carboydrate binding modules are involved in adhesion to carbohydrates and play an important role in the function of the cell wall in mycobacteria. The main structure of the cell wall is the mycolyl-arabinogalactan-peptidoglycan complex, which is made up of a variety of glycolipids that form an outer membrane that has low permeability and is crucial for the growth and virulence of Map and other mycobacteria [41]. CBM2 and CBM48 enhance the enzymatic activity of arabinofuranosyltransferase in the formation of the cell wall [42]. There was an additional CE10 CAZyme (part of the carbohydrate esterase class) present in Map Type C and B, carbohydrate esterase (carboxylesterase Ninh), is an alpha/beta hydrolase and have been identified in *M. tuberculosis* (Mtb). They are associated with metabolism and degradation of lipids, evasion and modulation of immune responses, detoxification, adaptations to growth, responses to acidification and dormancy [43]. In Mtb, these hydrolases have contributed markedly to the survival of this pathogen, and this may also be the case for Map, especially Type C Map which has adapted to a much larger species range than Type S Map and has been shown to be more virulent than Type S [1].

Secondary metabolites are compounds produced in metabolic pathways that are not essential to the function of the organism, however they can play an important role as metal transporting agents and aid in the survival of bacteria due to their antifungal, antibacterial and insecticidal properties [44]. Most of the secondary metabolites identified in Map in this study were glycopeptidolipids, including alkylresorcinol, isorenieratene and methylated alkyl-resorcinol/methylated acyl-phloroglucinol, all with > 70% gene similarity to the antiSMASH database. It is likely these molecules play a role in the cell wall of the bacterium, which is integral for the survival and pathogenesis of Map. There was only a small difference identified in the secondary metabolite profiles between Type C and Type S strains with Type C having an additional polyketide and non-ribosomal peptide synthetase cluster. The additional polyketide synthetase cluster in Type C strains was identified as PapA2 which is a polyketide synthase-associated protein that encodes virulence-enhancing lipids and is involved in cell adhesion to the host [7], which may also attribute to the increased virulence demonstrated in Type C strains due to their ability to infect multiple host species. The ‘methylated alkyl-resorcinol/methylated acyl-phloroglucinol’ cluster that has previously been reported as unique to Type C K-10 isolate [19] was identified in all Type C, Type S and Type B strains as well as the three Mac species analysed in this study with 100% identity to the antiSMASH database. These methylated polyketides are unique to Mycobacteria [45] and are an important part of the lipidic polyketides that make up the cell envelope and mediate infection in the pathogenic Mycobacterium [45]. The remaining nine secondary metabolite clusters identified (4 NRPS, 2 T1PKS, 2 NRPS.T1PKS, and a T1PKS) showed a low similarity to the antiSMASH database and their role in the biology of Map is unclear. Maa and Mah possessed one less PKS cluster than Map with the remaining secondary metabolite clusters being homologous to Map which may show the closer ancestral relationship between Map, Maa and Mah.

The total copy number of IS elements in Mac and Map ranged from 76–104 and 52–67 copies, respectively. The lower number of total copies of IS elements identified in the genomes of Map compared to Mac may be attributable to Map having a smaller environmental niche than most Mac species resulting in less environmental pressure. Two of the IS elements in Mah originated from *Rhodococcus*, whereas the origin of all the IS elements identified in the Map genomes were all from within the Mycobacterium sp., further highlighting the smaller environmental niche that Map occupies. There were no unique IS elements within the Map strain types, however Type S Map had a greater number of copies of IS900, IS1311, IS1245 and ISMgi2 (Additional file 5).

Integrated virus genomes or prophages are an important part of the bacterial genome playing crucial roles in the pathogenicity and survival of bacteria and adaptation to different ecological niches [46] and they contribute to some of the genetic variability found in individuals within a species [47, 48]. In this study there were six incomplete prophages identified in Map Type S of which an 8.1kb prophage region was found in the majority of Type S isolates and eight incomplete prophage regions identified in the Map Type C genomes, of which a 10.1kb and a 17.7kb prophage region was identified in the majority of the Type C isolates (Additional file 4). The 17.7kb prophage region which has previously been reported by Wibberg et el [19] and two putative prophage regions (LSP4 and LSP11) reported by Alexander et al. [13] are the only prophages that have previously been reported in Map. There were no complete prophages in any of the Map sequences identified in this study further confirming that Map has a closed genome and that remnants of the ancestral prophage are still there, but losses have occurred over time through genome rearrangements. There were no plasmids identified in any of the Map genomes analysed which is consistent with other studies [19]. Plasmids have been identified in some other mycobacterial species (eg. *M. tuberbulosis* and *M. abscess*) and are known for carrying pathogenicity-related genes, however Map has acquired different virulence systems (eg. ESAT-6) which have aided in their survival.

Clustered Regularly Interspaced Short Palindromic Repeats (CRISPR) are short repeat regions found in the DNA of many bacteria and archaea [49]. Cas genes (CRISPR-associated genes) are found in close proximity to the CRISPR and make up the CRISPR-cas defence system [50]. This defence system is said to be horizontally transferred by transduction [51] via bacteriophages that had once infected the host organism providing a form of acquired immunity [52]. In this study the cas associated genes were not found in any of the Mac or Map genomes, indicating that this defence system has not been acquired by these Mycobacterial species. There were several different CRISPR sequences identified in both Mac and Map, however there were no CRISPR sequences that were unique to any of the subspecies of Mac or within the Map strain types. To date both *M. bovis* and *M. tuberculosis* have been identified as the only two mycobacteria species to contain the CRISPR-cas defence system [53]. As both Mac subspecies and Map contain some CRISPR regions but no cas associated genes it is possible that they have been lost over the course of evolutionary history as they are no longer required and Mac species use alternative defensive systems such as ESX-1 and ESAT-6, that are essential for virulence and are involved in macrophage escape by Mycobacteria [29].

### The ancestral lineage of Map

Over the last 20 years evolutionary relationships for Mac and Map have been proposed using data from 16s sequencing, multilocus sequence analysis (MLSA), and more recently small-scale genome sequence studies [20, 35, 52, 53]. The ancestral lineage of Maa and Map within Mac has been suggested to be derived from *M. avium* subsp. *hominissuis* [43, 53]. By using pulse field gel electrophoresis (PFGE) Map was further subdivided into Type S and Type C with the hypothesis that Map Type C emerged from Type S [54]. Phylogenetic core genome analysis and the homologue matrix analysis of 268 Mac and Map sequences conducted in this study has further confirmed this ancestral lineage of Maa and Map. Structural comparative analysis of the Map strains suggest that the Type S strains of Map were more likely to have emerged before Type C and Type B strains. The Type S strains had a greater variation in gene synteny when compared to Type C strains which was evident from the larger number of genetic rearrangements [55] observed in Type S Map. The monomorphic nature of the Type C strains was also demonstrated by a previous phylogenetic SNP analysis [6] and the core gene analysis in this study that showed little diversity in the branches of the phylogenetic tree of Type C strains. Pan genome analysis showed that there is greater genetic diversity within Type C strains as they have a much larger pan genome size and more accessory genes than Type S. Cattle, the predominant host of Map Type C isolates, were originally used for draught and transport because of their size and strength [56]. They were used for cultivating the land, pulling heavy loads, pulling milk wagons, cars, school buses, fire engines amongst others and they always worked in small groups [57]. The use of cattle as draught power meant they were mixing with other cattle and other animal species within and between communities and in turn exposed to a potentially broader environmental niche. This may account for the larger accessory genome in Type C strains which were likely a result of gene acquisition via horizontal gene transfer as several of the Type C accessory genes were derived from other bacteria. For example, some incomplete prophages were identified as a *Rhodococcus* and *Streptomyces* prophage in both Type C and Type B genomes whereas prophages identified in Type S genomes were all identified as Mycobacterial prophages. The first known wild sheep (Siberian Bighorn and bighorn sheep) mostly wandered in small groups of 8–10 and inhabited mountainous areas and rugged cliffs [58] where most other animals could not inhabit so they were not exposed to many other animal species which would account for the smaller pan genome of Type S Map compared to Type C. A higher number of regions of genome plasticity were also identified in Type C genomes when compared to Type S strains indicating that Type C strains were further evolving from Type S strains as they became exposed to a larger external gene pool. Previous core SNP analysis also showed similar topology of Map to the core gene analysis in this study showing the ancestral lineage of Type C emerging from Type S [6].

There was little to no differences found between the genomes of Type B and Type C Map; there were no differences in IS elements, prophages, secondary metabolites, CAZymes, CRISPr elements and little to no structural differences between Type C and Type B Map. A previous study [6] found no difference in ANI and found there were no long sequence polymorphisms (LSP’s) that can differentiate between Type C and Type B Map. Type B Map has been differentiated based on using the IS1311 PCR and REA [3]. However this approach has previously been shown to be unreliable for differentiating between the Map strain types [6]. As this study has identified that Type B and Type C map are structurally similar and have little to no differences between their genomes it is proposed that Type B is not a different strain type of Map but a distinct clade within Type C Map strain.

## Conclusion

This study represents the most comprehensive comparative genomic analysis of Map strains and has improved our knowledge on the genomic and structural differences between Mac, Map and the subtypes of Map. Genomic analyses has confirmed the ancestral relationship between Mah and Map and the Type S and Type C strains within Map and improved our understanding of the differences between the subtypes of Map and the genetic diversity within Type S strains.

This analysis also supported previous theories of the ancestral lineage of Map, with the Type C strain emerging after the Type S strain [59, 60]. Mauve alignments showed more genome rearrangements within Type S strains indicating greater genetic synteny in Type S strains when compared to Type C strains. Pan-genome analysis shows that Type C strains have a larger accessory genome than Type S strains indicating that the greater genomic diversity within this strain type is mostly likely due to cattle being exposed to a larger gene pool than sheep. There was also little to no genetic variation between Type C and Type B Map strains indicating that Type B Map isolates may be a distinctive clade within the Map Type C strain group rather than a different strain type of Map.

The identification of the unique core genes in Map and Type S strains in this study will lead to the development of more highly specific diagnostic tools for the identification of Map and more rapid tools for strain typing of Map. The information gained from the analysis performed in this paper will also contribute to further research into the functional genomics of Map.

## Methods

### Panel of strains and Illumina sequencing

A total of 211 Map isolates and one *M. avium* subsp. *avium* isolate from the Australian Johne’s Disease Reference Collection (AJDRC) that had previously been sequenced [6] were selected for pan-genome analysis in this studybased on a selection of Type C, Type S and Type B strains of Map. A further 8 Map strains obtained from the Moredun Research Institute, England were sequenced; DNA extraction and sequencing was performed as previously described [6]. Twenty eight Map strains from diverse geographical regions [61, 62] and 4 subspecies within the Mac (*Mycobacterium avium subsp. avium*, accession NZ_CP016396, *Mycobacterium chimaera* AH16, accession PRJNA294790; *Mycobacterium intracellulare*, accession CP023149 and NC_016946; *Mycobacterium avium* subsp. *hominissuis*, accession CP040247 and NC_008595) were downloaded from the European Nucleotide Archive database and the SRA database from the National Center for Biotechnology Information (NCBI). An additional 14 complete Map genomes were also downloaded from NCBI (Map K-10, accession NC_002944.2; Telford strain, accession NZ_CPO33688.1; MAPK_JJ1/13, accession NZ_CP033909; MAPK_JB16/15, accession NZ_CP033911; MAPK_CN9/15, accession NZ_CP033427; MAPK_CN7/15, accession NZ_CP033428; MAPK_CN4/13, accession NZ_CP033910; JIII-386, accession CP042454; JII-1961, accession NZ_CP022105; and FDAARGOS_305, accession NZ_CP022095, E1, accession CP010113; E93, accession CP010114; MAP4, accession CP005928 and Tn-India, accession CP015495). Only draft genome sequences of isolates that were assembled from less than 200 contigs were used in this study. 

### Nanopore Library Preparation and MinION® sequencing and analysis

Long read sequencing of six selected Map genomes from the AJDRC was conducted to generate closed genomes of these isolates. Nanopore libraries from genomic DNA were prepared using the nanopore sequencing kit SQK-LSK109 (Oxford Nanopore Technologies, UK) with EXP-NBD104 barcode kit (Oxford Nanopore Technologies, UK) according to the manufacturer’s instructions with some modifications. ProNex® Size-Selective Purification System (Promega, Madison, WI, USA) beads were used for end repair and native barcode ligation. Sequencing was performed on an Oxford Nanopore MinION Mk1b sequencer, using an R9.4.1 flow cell that was prepared according to the manufacturer’s instructions. MinKNOW was used to control the run using the 72h sequencing run protocol. Basecalling and demultiplexing was performed offline using Guppy (v2.2.3) [63] which is a data processing toolkit that contains the Oxford Nanopore Technologies’ basecalling algorithms. Nanoplot (v1.33.0) [64] was used to check the quality of the long read sequencing data produced by the MinION sequencing. The assemblies were then visualised in Bandage (v0.8.1) [65] to check for a complete circular contig. De novo assembly was performed using the MinION quality trimmed long reads and the Illumina quality trimmed short reads using the hybrid assembly method of Unicycler (v0.4.7) [66] to produce a complete genome assembly.

### Genome construction

Quality trimming and genome assembly was performed as previously described [6]. Quast version 5 [67] was used to assess the quality of all the assemblies using the features flag. All 268 assemblies (including the eight complete genomes from MinION sequencing) were annotated using Prokka version 1.14.5 [68] using the genus and addgenes flags.

### Analysis of genome content

In this study draft genomes that had greater than 200 contigs were excluded from the analysis to avoid the possibility of having fragmented genes on the contig boundaries and misassembles which may result in an over representation of accessory genes and an under representation of core genes [69].

ANI of all possible pairs of assembled genomes was calculated as previously described [6].The annotated assemblies of the 268 Map and Mac genomes in gff format were used to perform pan genome analysis using Roary version 3.11.2 [70]. The core gene alignment was then used to create a phylogenetic tree using RAxML version 8.2.11 [71], which was then annotated using FigTree version 1.4.4 [72]. A Roary plot script written by Marco Galardini available within the Roary package was used to create the pan-genus homologue matrix and pan-genome pie plot. The gene presence/absence data was further interrogated using the query-pan genome script also available within the Roary package. Statistics called by the pan-genome analysis defined the core genes as being present in 99–100% of strains, soft core genes in 95–99% of strains, shell genes in 15–95% of strains and cloud genes in 0–15% of strains. A pan-genome database of protein sequences was generated by compiling all the sequences of Map and Mac. To confirm the identify of genes that were only present in all the Map genomes and those that were only present in all the Mac genomes a query set of protein sequences was created by compiling all these genes together. This query set was then blasted against the database using an E value of 10^–25^ as a cut-off threshold and > 90% similarity value. Further confirmation of the unique genes that had been identified in Mac and the Map strain types was performed using Geneious Prime version 2022.0.2, (https://www.geneious.com). Briefly, all the unique genes that had been identified were mapped back to the reference genomes (Type C K-10 Map, Type S Telford, and Type B M107/05 field isolate). A fasta file containing all the coding sequences (CDS) for these unique genes was compiled and their functional categories were assigned using DIAMOND in egg-nogg mapper [73]. These functional categories were further grouped into four classes: Metabolism; Information, storage and processing; Cellular processes and signalling; and poorly characterised. A query set of protein sequences that were only present in each of the Map strain types was also created and blasted against the database to confirm the identify of those genes.

### Structural comparative analysis of genomes

The 25 complete genomes analysed in this study were aligned to each other using the multiple genome alignment system Mauve 2.4.0 [74] using the progressive Mauve algorithm to identify the presence of evolutionary events such as rearrangements and inversions.

The software PPanGGolin version 1.1.136 [75] was used to identify regions of genome plasticity (RGP) in the dataset. The RGP for each isolate were extracted using bedtools and converted to a single fasta file. BLAST Ring Image Generator (BRIG) [76] was performed on 48 isolates based on a selection of Type S Map, Type B Map and Type C Map (Additional file 12) that phylogenetically belonged to different clades [6]. Genomic regions of interest were then viewed on Artemis Comparison Tool (ACT) [77] to identify the genes and their associated function was determined using blastp [63].

### Secretory systems and effector proteins

Nucleic acid sequences of the genes associated with secretory systems and effector proteins that were identified from previous published data [19, 78] and from the Virulence factors Database, Mycobacterium (VFDB, http://www.mgc.ac.cn/cgi-bin/VFs/genus.cgi?Genus=Mycobacterium) were compiled together to create a query set of secretory system genes and effector proteins. This query set was then blasted against the previously established Map and Mac database using an E value of 10^–25^ as a cut-off threshold and > 90% similarity value to confirm their identity in the Map and Mac genomes. The function of each of the effector proteins was confirmed using protein BLAST and NCBI’s conserved domain database [79, 80].

### Carbohydrate active enzyme profiles (CAZyme) identification

Coding sequences of the carbohydrate active enzymes within each genome were identified using HMMER version 3.2.1 [81] against the dbCAN database [82] with an e-value less than 1e^−17^ and greater than 35% query coverage. The CAZyme hits were then clustered and the CAZyme profiles of 35 representative genomes were plotted as a cluster matrix and dendogram using pheatmap version 1.0.12 [83] in R version 4.1.0 [84].

### Secondary metabolite

The bacterial version of antiSMASH 5.1.0 [85] was used to identify secondary metabolite biosynethesis gene clusters in the 25 complete genomes analysed in this study.

### Insertion sequences

IS Finder (http://www-is.biotoul.fr) [86] was used to identify Insertion sequences in all 268 genomes with a bit-score > 50 and an E-value of 10^–5^ used as a cut-off threshold. IS element protein sequences were added to the query set of protein sequences used for the identification of unique genes as described above. The IS elements identified using IS Finder were then confirmed and the number of copies identified by blasting this query set against the pan-genome database with a cut-off of > 75% similarity value.

### Integrated prophages and prophage-like elements

PHASTER (PHAge Search Tool Enhanced Release) [87] was used to identify and annotate prophage sequences within all the 268 genomes. The identified prophage sequences were then blasted against the Actinobacteriophage Database (http://phagesdb.org/blast/) to identify any significant prophage matches within the database.

### Plasmid identification

PlasmidSpades (version 3.15.3) [88] was used to identify plasmid DNA in all of the 268 genomes. Blastn [79] was then used to confirm the identification of the plasmids.

### Clustered Regularly interspaced Short Palindromic Repeats (CRISPRs)

CRISPRs were identified in all the Map and Mac genomes using CRISPRCasFinder version 4.2.20 [89]. A CRISPR database of all the nucleotide sequences of the identified CRISPRs was generated. A FASTA file was then created of all the unique CRISPRs to be used as the query set of nucleotide sequences. This query set was then blasted against the database using an E value of 10^–25^ as a cut-off threshold and > 90% similarity value. 

### Supplementary Information

**Additional file 1.**

**Additional file 2.**

**Additional file 3.**

**Additional file 4.**

**Additional file 5.**

**Additional file 6.**

**Additional file 7.**

**Additional file 8.**

**Additional file 9.**

**Additional file 10.**

**Additional file 11.**

**Additional file 12.**

## Data Availability

The datasets generated and/or analysed during the current study are available in the NCBI Bioproject repository, accession number PRJNA632696 (https://www.ncbi.nlm.nih.gov/sra/PRJNA632696).
