# Supplementary material for: The pan-genome of Mycobacterium avium subsp. paratuberculosis (Map) confirms ancestral lineage and reveals gene rearrangements within Map Type S
Source: BMC Genomics. 2023 Oct 31;24:656. doi: 10.1186/s12864-023-09752-0 (PMC10619280; doi:10.1186/s12864-023-09752-0)
Supplement: Supplementary file 8 — Additional file 8. [file 12864_2023_9752_MOESM8_ESM.docx]

**Additional File 8.** CAZyme metadata of each family identified in Map according to the CAZY database (<http://www.cazy.org>) including activities in family, prokka annotation and gene name

| **CAZyme Family** | **Activities in family** | **Prokka Annotation** | **Gene Name** |
| --- | --- | --- | --- |
| **Carbohydrate-Binding Modules (CBMs): adhesion to carbohydrates** | | |  |
| CBM2 | Modules of approximately 100 residues and which are found in a large number of bacterial enzymes. The cellulose-binding function has been demonstrated in many cases. Several of these modules have been shown to also bind chitin or xylan | Putative protein | Group_3209 |
| CBM48 | Modules of approximately 100 residues with glycogen-binding function, appended to GH13 modules. Also found in the beta subunit (glycogen-binding) of AMP-activated protein kinases (AMPK) | Glycogen operon protein Glgx | glgX_1 |
| **Carboyhydrate Esterases (CEs): hydrolysis of carbohydrate esterases** | | |  |
| CE1 | acetyl xylan esterase (EC 3.1.1.72); cinnamoyl esterase (EC 3.1.1.-); feruloyl esterase (EC 3.1.1.73); carboxylesterase (EC 3.1.1.1); S-formylglutathione hydrolase (EC 3.1.2.12); diacylglyerol O-acyltransferase (EC 2.3.1.20); trehalose 6-O-mycolyltransferase (EC 2.3.1.122). | Hypothetical protein, Diacylglycerol acyltransferase/mycolyltransferase Ag858 | Group_4374, fbpB |
| CE10 | arylesterase (EC 3.1.1.-); carboxyl esterase (EC 3.1.1.3); acetylcholinesterase (EC 3.1.1.7); cholinesterase (EC 3.1.1.8); sterol esterase (EC 3.1.1.13); brefeldin A esterase (EC 3.1.1.-) | Para-nitrobenzyl esterase | pnbA_1 |
| CE14 | N-acetyl-1-D-myo-inosityl-2-deoxy-α-D-glucopyranoside deacetylase (EC 3.5.1.89); diacetylchitobiose deacetylase (EC 3.5.1.-); mycothiol S-conjugate amidase (EC 3.5.1.-) | 1D-myo-inositol 2-acetamido-2-deoxy-alpha-D-glucopyranoside deacetylase | mshB |
| CE4 | acetyl xylan esterase (EC 3.1.1.72); chitin deacetylase (EC 3.5.1.41); chitooligosaccharide deacetylase (EC 3.5.1.-); peptidoglycan GlcNAc deacetylase (EC 3.5.1.-); peptidoglycan N-acetylmuramic acid deacetylase (EC 3.5.1.-) | Hypothetical protein | Group_5433 |
| CE5 | acetyl xylan esterase (EC 3.1.1.72); cutinase (EC 3.1.1.74) | Putative cutinase cut2 | Cut3_2 |
| CE9 | N-acetylglucosamine 6-phosphate deacetylase (EC 3.5.1.25); N-acetylgalactosamine 6-phosphate deacetylase (EC 3.5.1.80) | Hypothetical protein | Group_279 |
